# Bioinformatics Approaches for Functional and Structural Annotation and Molecular Docking Study of a Hypothetical Protein From *Staphylococcus aureus*

**DOI:** 10.1155/bmri/1109584

**Published:** 2026-06-07

**Authors:** Mehedi Hasan Chowdhury, Md. Reaz Morshed, Mohammad Jane Alam, Sazzad Hossain, Hasna Hena

**Affiliations:** ^1^ Department of Biochemistry and Molecular Biology, Noakhali Science and Technology University, Noakhali, Bangladesh, nstu.edu.bd

**Keywords:** HAD IIIA-type phosphatases, homology modeling and molecular docking, hypothetical protein (ohs92176.1), *Staphylococcus aureus*

## Abstract

*Staphylococcus aureus* is a common Gram‐positive bacterium that colonizes the skin and mucous membranes of humans. It is a major etiological agent of nosocomial infections and a persistent source of contamination in food and hospitals. The complete sequencing of the *S. aureus* genome has been accomplished, yet numerous hypothetical proteins encoded within it remain functionally uncharacterized. For this study, we performed a comprehensive functional and structural analysis of a hypothetical protein (accession number OHS92176.1) from the *S. aureus* HMSC77A05 strain using bioinformatics approaches. Physicochemical investigations showed that the protein is stable, soluble, and cytosolic. Functional annotation using NCBI Conserved Domain Search, InterProScan, and Pfam databases identified that the protein is a member of the YqeG family of HAD IIIA‐type phosphatases within the haloacid dehalogenase (HAD) superfamily. Multiple sequence alignment showed that many strains of *S. aureus* are very similar in their evolution, indicating that they are likely important for their function. Both PSIPRED and SOPMA consistently predicted that *α*‐helical elements are the most common part of the secondary structure of proteins. A three‐dimensional model was constructed using SWISS‐MODEL and template A0A2C6WKT6.1.A, exhibiting 71.43% sequence identity with the target protein. The model quality underwent validation using ERRAT, Verify3D, QMEAN, and PROCHECK analyses, all of which affirmed its structural reliability and stereochemical accuracy. STRING′s study of protein–protein interactions found associations with enzymes involved in ribosomes, amino acid biosynthesis, and nucleotide catabolism. It was also discovered that the active site has four putative catalytic pockets. Molecular docking simulations were subsequently conducted using six phytocompound ligands—quercetin, kaempferol, naringenin, apigenin, catechin, and curcumin—all of which demonstrated favorable binding affinities. Notably, kaempferol exhibited the highest binding affinity (*ΔG* = −8.3 kcal/mol). These findings indicate that this previously uncharacterized hypothetical protein may serve as a promising therapeutic target for an antibacterial agent against *S. aureus*. The comprehensive computational analyses provide a robust foundation for subsequent experimental validation and structure‐based drug development.

## 1. Introduction

In modern proteomics research, understanding the structural and functional attributes of proteins is essential for the creation of novel therapeutic agents. A thorough analysis of the proteome is crucial for identifying new therapeutic targets in bacterial pathogens, especially given that a significant number of proteins, known as hypothetical proteins (HPs), are still functionally and structurally uncharacterized. These proteins are predicted based on open reading frames but lack empirical confirmation of their expression or function [1–3]. These proteins act as an unexplored reserve of significant biomolecules that may contribute significantly to biochemical processes and pathogenicity. Functional annotation of such proteins is therefore essential for improving our understanding of bacterial biology and for identifying new therapeutic targets [3]. Among various enzyme families, the haloacid dehalogenase (HAD) superfamily constitutes a diverse group of phosphatases involved in essential metabolic and regulatory pathways [4, 5]. These enzymes are known to be implicated in phosphate metabolism and other important cellular processes, which make them good candidates for finding new antimicrobial drugs [6].

However, many HAD family proteins in *Staphylococcus aureus* remain poorly characterized. *S. aureus* is classified as a gram‐positive bacterium and a major human pathogen that causes various types of infections, including mild skin and soft tissue infections, as well as acute, life‐threatening illnesses impacting the heart, lungs, bones, blood, and other organs [7, 8]. In addition, *S. aureus* possesses numerous virulence factors and undergoes mechanisms that enable it to adapt to various host environments and exhibit antibiotic‐resistant strains, especially methicillin‐resistant *S. aureus* (MRSA), which complicates treatment options and increases the urgency for new therapeutic strategies. The increasing prevalence of MRSA strains highlights the urgent need to explore previously uncharacterized proteins as potential targets for drug or vaccine development [8, 9]. Despite the availability of complete genome sequences from prior research, our understanding of numerous HPs in *S. aureus* remains incomplete. The limited understanding of these proteins impedes a comprehensive comprehension of their roles in biological systems, as well as their involvement in disease development and the emergence of antibiotic resistance [10, 11]. A more thorough understanding of HPs′ structures and functions could facilitate the identification of new biomarkers and drug targets, thus expediting the development of novel antimicrobial therapies [12].

Furthermore, recent progress in computational biology has enabled the application of bioinformatics techniques to provide functional and structural annotations for HPs [13]. The advent of precise protein structure prediction techniques, exemplified by AlphaFold, has significantly improved the dependability of computational structural analyses, especially for proteins whose structures have not been experimentally resolved [14]. The computational tools are able to predict three‐dimensional (3D) structures, conserved domains, motifs, biological processes, and networks of protein–protein interactions (PPIs) [15, 16]. Furthermore, structure‐based molecular docking (MD) has demonstrated its utility in identifying potential inhibitors and facilitating the early stages of drug development [17]. The *S. aureus* strain HMSC77A05, isolated from a human host, has a genome that is about 2.8 Mb in size and contains 2678 protein‐coding genes. The GC content is 32.5%. Of these, 304 proteins, or about 11.36%, are classified as “hypothetical” [18]. Among these, hypothetical protein OHS92176.1 from *S. aureus* was selected because of its conserved domain architecture and predicted classification within the YqeG family of HAD phosphatases, which is essential for cellular metabolic processes. The potential involvement in essential metabolic pathways and its possible importance as a therapeutic target make it a captivating candidate for structural and functional characterization.

Employing a multifaceted bioinformatics strategy encompassing physicochemical assessment, domain prediction, homology modeling, phylogenetic analysis, PPI network analysis, and MD, we investigated the functional and structural attributes of OHS92176.1. The objective of this investigation is to establish a link between protein annotation and the identification of therapeutic targets, thereby characterizing OHS92176.1 as a potential candidate for the development of novel antimicrobial agents.

## 2. Methods and Materials

**Figure 1:**
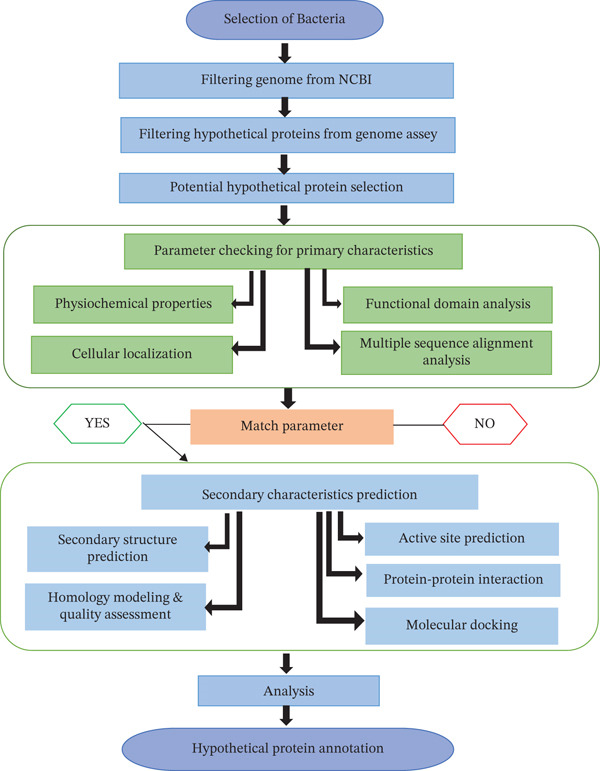
The complete schematic representation of the hypothetical protein annotation process utilized in the experiment.

The overall workflow executed for the functional annotation and structural characterization of the hypothetical protein from *S. aureus* is depicted in Figure 1.

### 2.1. Protein Sequence Retrieval

The HPs of *S. aureus* were searched in the NCBI protein database. An HP (accession no. OHS92176.1) from *S. aureus* was selected for this study, which contains 175 amino acid (AA) residues. The primary sequence of the HP was obtained in the format of FASTA to conduct further bioinformatics studies [19].

### 2.2. Analysis of Physicochemical Characteristics

ProtParam software, accessible on the ExPASy platform (http://web.expasy.org/protparam/), was utilized to forecast physical and chemical properties of the targeted HP. This bioinformatic tool provides a rapid and efficient approach to obtain thorough insights into molecular weight (MW), extinction coefficient, composition of AAs, and potential isoelectric point (pI) of the protein. The information allows for the prediction of the structure, function, and stability of the protein. ProtParam is capable of calculating both the instability index (II) and aliphatic index (AI) of the HP. The stability across diverse environs and the solubility in water of the HP can be forecasted through these indexes. These are useful predictions in designing the experiments and producing new drugs [20].

### 2.3. Prediction of Intracellular Distribution and Solubility

The intracellular distribution of the HP was anticipated by the CELLO v.2.5 program (http://cello.life.nctu.edu.tw/), which utilizes a support vector machine (SVM)–based classification system that incorporates sequence‐derived features, including AA composition and physicochemical properties [21, 22]. The Protein‐Sol server (https://protein-sol.manchester.ac.uk/) was employed to figure out the solubility of targeted HP in contrast to a population average solubility score (PopAvrSol) derived from experimentally validated *Escherichia coli* expression datasets [23].

### 2.4. Investigation of Functional Domain and Family/Superfamily

HPs are categorized into families and subfamilies based on sequence features, conserved domain structure, and functional homology using both manual and bioinformatics techniques. The NCBI Conserved Domain Database (CDD) Search tool (https://www.ncbi.nlm.nih.gov/) and the InterProScan tool (https://www.ebi.ac.uk/interpro/search/sequence/) were commonly utilized to evaluate conserved domain and functional motifs within the sequence of targeted HP [24, 25]. The CDD analysis was performed using CD‐Search with default parameters and an *E*‐value threshold to find statistically significant domain matches against curated domain models. Position‐specific scoring matrices can identify conserved structural and functional domains [24].

Using default settings, InterProScan analysis integrated Pfam protein signature databases to give domain annotation and functional classification [25].

### 2.5. Analysis of Multiple‐Sequence Alignment (MSA) and Tree of Phylogeny

A BLASTp search was performed utilizing NCBI′s unique protein repository (http://www.ncbi.nlm.nih.gov/) with default parameters to identify homologous protein sequences [26]. Subsequently, MSA was generated and analyzed utilizing Jalview, a robust bioinformatics platform designed for alignment visualization and analysis [27]. Jalview facilitates the integration of external tools for constructing phylogenetic trees and allows for the visualization and manipulation of these trees in conjunction with aligned sequences. Phylogenetic analysis was conducted utilizing the generated MSA, and a phylogenetic tree was constructed employing the neighbor‐joining (NJ) method with 1000 bootstrap replicates to guarantee the robustness and reliability of the inferred evolutionary relationships [28, 29].

### 2.6. Analysis of Two‐Dimensional (2D) Structure

To detect the 2D structure of the HP, the PSIPRED server (http://bioinf.cs.ucl.ac.uk/psipred/) and the SOPMA tool (https://npsaprabi.ibcp.fr/cgibin/npsa_automat.pl?page=/NPSA/npsa_sopma_f.html) were utilized [30, 31]. In predicting the three‐state secondary structure—which comprises alpha helices, beta sheets, and coils—SOPMA, a popular tool for protein secondary structure prediction, attains an accuracy of about 69.5% [31].

### 2.7. Homology Modeling and Tertiary Structure Analysis

Using the SWISS‐MODEL program (https://swissmodel.expasy.org/), homology modeling had been employed for determining the target protein′s tertiary structure [32]. Template identification was performed using an internal BLASTp search against available structural databases to identify suitable homologous structures for the input sequence. Because of the lack of experimentally resolved structures exhibiting high sequence similarity in the Protein Data Bank (PDB), a template based on AlphaFold v2 predictions was chosen for the model construction. The 3D structure of the target protein was therefore anticipated using AlphaFold v2 via the SWISS‐MODEL platform. In particular, template A0A2C6WKT6.1.A from *Staphylococcus edaphicus* was selected for model construction due to its favorable alignment characteristics. Templates derived from AlphaFold have shown the ability to deliver structural predictions with high accuracy, often matching the results obtained through experimental techniques in numerous instances [26]. The selected template revealed a sequence identity of 71.43%, a sequence coverage of 93%, and a Global Model Quality Estimation (GMQE) value of 0.93 with the target protein, indicating a strong degree of accuracy and reliability for precise tertiary structure prediction [32].

### 2.8. Quality Assessment

The stereochemical characteristics and structural righteousness of the modeled protein were assessed using the SAVES v6.0 server (https://saves.mbi.ucla.edu), which incorporates various validated bioinformatic tools [33–35]. Structural validation was conducted via PROCHECK to evaluate backbone dihedral angles by Ramachandran plot analysis [33], Verify3D to test the congruence of the 3D model with its AA sequence [34], and ERRAT to examine non‐bonded atomic interactions [35].

The overall quality of the predicted model was assessed utilizing the QMEAN4 scoring tool provided by the SWISS‐MODEL workspace located on the ExPASy server (https://swissmodel.expasy.org/qmean/) [36]. Additionally, the ProSA‐Web server was utilized to assess the overall quality of the protein model by contrasting it with experimentally derived structures. The ProSA‐Web tool assesses a z‐score that reflects the similarity between the input structure and a database of high‐quality, empirically determined protein structures, thus facilitating the identification of potential errors [37].

### 2.9. Analysis of Protein–Protein Interrelation Network

A protein interaction network analysis was performed to identify significant PPIs of investigated HP utilizing the STRING database version 12 (https://string-db.org). The STRING database methodically compiles and integrates PPIs, encompassing both physical (direct) interactions and functional (indirect) associations. The data are derived from multiple sources, including automated text mining of scientific literature, computational predictions of interactions based on co‐expression, conserved genomic context, and databases of interaction experiments and established complexes/pathways from curated sources [38, 39].

### 2.10. Identification of the Active Site

The PrankWeb server [40] was utilized for analyzing and comprehending the 3D structure of proteins. It is especially efficient in detecting and analyzing concave regions on protein surfaces, also known as pockets and voids. The binding of a substrate, inhibitor, or ligand molecule might implicate these regions in protein function. This provides detailed information about these pockets and voids, including their size, shape, volume, and surface area [41]. This information helps researchers to understand how such regions would interact with other molecules and potentially influence protein activity. Pocket ID is a fast and informative way to define and differentiate pockets in a protein structure, aiding studies in comprehending protein function, drug design, and protein engineering [42].

### 2.11. MD Analysis

MD analysis was conducted by utilizing the Autodock‐Vina tool (http://vina.scripps.edu/download.html). AutoDock‐Vina serves as a virtual screening and MD program that employs multithreading capacities on multicore platforms to enhance the precision of ligand–protein binding forecasts [43]. The docking simulation was performed using the PyRx platform, which incorporates AutoDock Vina to evaluate the potential interactions between the selected ligands and the HP. The six natural compounds—quercetin, kaempferol, naringenin, apigenin, catechin, and curcumin—were selected as potential ligands due to previous literature showing their inhibitory activities on *S. aureus* and associated bacterial strains [44–48]. The initial phase of conducting the docking study involved the meticulous preparation of the HP and ligands. The protein structure was meticulously prepared using the AutoDock tool, involving the removal of water molecules and other heteroatoms, followed by the addition of polar hydrogen atoms and partial Kollman charges. Energy minimization was used to reduce steric conflicts and optimize protein geometry. The protein was subsequently saved in a format compatible with AutoDock, utilizing the PDBQT file extension for the docking study. The ligand molecules were energy‐minimized and translated into PDBQT format, with Gasteiger charges applied. A grid box was made by choosing the AAs that make up the protein′s active site in order to make the configuration file. The following settings were used to set up the grid box in AutoDock Vina: The *X*‐dimension is 44.6349 Å, the *Y*‐dimension is 47.4573 Å, and the *Z*‐dimension is 42.5865 Å. The grid center′s coordinates were set to *X* = 0.4760, *Y* = −3.6884, and *Z* = −2.4844. The exhaustiveness parameter was set at 8 to make sure that the conformational sampling was good enough. The BIOVIA Discovery Studio 2024 was utilized to visualize and investigate the docking results from AutoDock Vina, which included binding affinities and interaction residues between the prepared ligand and HP.

## 3. Results

### 3.1. Physicochemical Features of the Protein

The ProtParam tool was utilized in order to conduct an analysis of the physicochemical properties of the HP (OHS92176.1) (Table 1 **)**. The HP comprises 175 AAs, possesses an MW of 20,171.45 Da, and an isoelectric point (pI) of 9.73, indicating it is basic in nature. The grand average of hydropathicity index (GRAVY) is −0.298, demonstrating a hydrophilic nature that actually enhances better interactions in an aqueous cellular environment.

**Table 1:** Physicochemical attributes of the studied HP OHS92176.1 obtained by executing the ProtParam tool.

Parameter	Value
Number of AAs	175
MW (Da)	20,171.45
PI	9.73
Negatively charged residues (aspartate + glutamate)	21
Positively charged residues (arginine + lysine)	29
Projected duration of half‐life	30 h (mammal reticulocytes, in vitro) Greater than 20 h (Yeast, in vivo) Greater than 10 h (*E. coli*, in vivo)
II	29.61 (Stable)
AI	90.11
GRAVY	−0.298 (Hydrophilic)

The II is calculated as 29.61, suggesting that the annotated HP exhibits high thermostability.

Moreover, the greater number of positively charged residues (29) in contrast to negatively charged ones (21) buttresses its basic nature. The predicted half‐life of the HP indicates that it maintains a considerable degree of stability across various biological systems.

### 3.2. Intracellular Distribution and Soluble Nature

**Figure 2:**
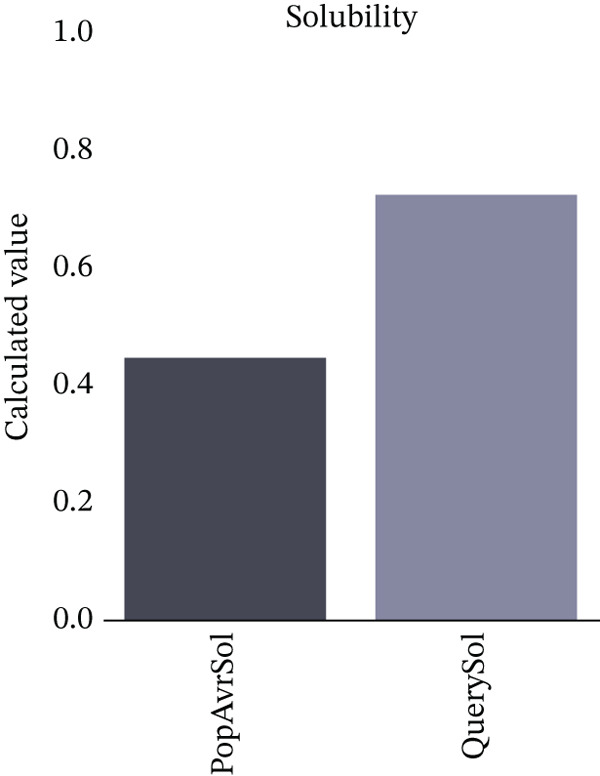
Comparison of the predicted solubility of the QuerySol with the PopAvrSol.

The exact position of a protein within a cell can be identified by detecting its subcellular localization. The CELLO v2.5 program uncovered that the examined HP is predominantly compartmentalized in the cytoplasm, indicating that it plays roles in intracellular processes (Table 2). The Protein‐Sol server did the protein solubility analysis. It also indicated that the HP is soluble because the QuerySol value for the query protein was about 0.7, which is much higher than the population average solubility (PopAvrSol) of about 0.45. (Figure 2). The results suggest that the protein is probably capable of retaining solubility in physiological conditions. The estimated solubility scores are represented on the *y*‐axis, with a scale from 0.0 to 1.0.

**Table 2:** Analysis of the subcellular localization of the HP OHS92176.1 from *S. aureus as* determined by the CELLO v2.5 server.

Methods	Features	Predicted localization	Reliability/score
SVM analysis	AAs, N‐peptide, partitioned sequence, physicochemical, and neighboring sequence composition	Cytoplasmic	0.616
			0.875
			0.687
			0.646
			0.454
CELLO prediction	Cytoplasmic	3.279 ^∗^	
	Periplasmic	0.673	
	Outer Membrane	0.479	
	Inner Membrane	0.303	
	Extracellular	0.267	

### 3.3. Analysis of Functional Domain and Family/Superfamily

The NCBI‐CDD, Pfam, and InterProScan programs were used to perform a conserved domain study of the HP. The NCBI CD Search identified a YqeG family HAD IIIA‐type phosphatase domain, which is part of the HAD superfamily. This domain spans AA positions 6 to 168 and has a highly significant *E*‐value of 1.48e‐86. It may be involved in the proper formation of colonies, as seen with *Bacillus subtilis* YqeG proteins, and it is homologous to phosphatases that hydrolyze phosphate groups from 5 ^′^‐nucleotides. Furthermore, the Pfam database identified a conserved HAD‐like domain spanning the AA range of 7 to 170, with an *E*‐value around 1e‐70, which further substantiates the classification of the HP within the HAD superfamily.

### 3.4. Analysis of MSA and Phylogenetic Tree

**Figure 3:**
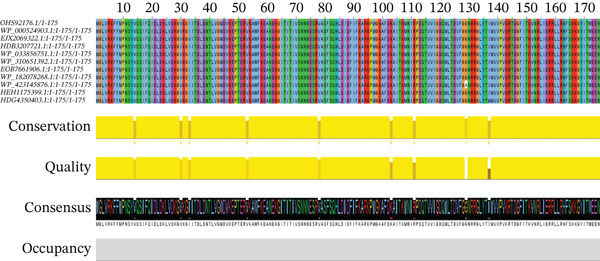
Multiple‐sequence alignment study of the HP OHS92176.1 and other YqeG family HAD IIIA‐type phosphatase proteins. The alignments were conducted utilizing Clustal Omega as integrated within Jalview version 2.11.1.3. Functionally significant residues and conserved areas are highlighted in the alignment. The homologous sequences from *Staphylococcus* species are displayed after the target HP OHS92176.1 sequence in the first row. In this context, Row 2 illustrates the sequence of WP_000524903 (*Staphylococcus* spp.), whereas Rows 1, 3, 4, 5, 6, 7, 8, 9, 10, and 11 respectively correspond to the sequences of OHS92176.1, EJX2069322, EOB7661906, HDB3207721, WP_033856751, WP_310651392, WP_182078268, HEH1175399, WP_423145876, and HDG4350403 (*S. aureus*).

**Figure 4:**
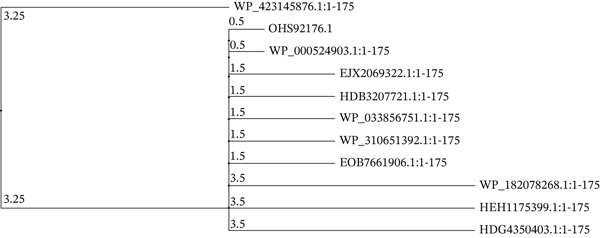
Phylogenetic relationships among the targeted HP OHS92176.1 and other similar proteins. The evolutionary distances in a phylogenetic tree were determined through the application of the neighbor‐joining (NJ) method, relying on pairwise distance calculations derived from the aligned sequences. The nodes display bootstrap values obtained from 1000 replicates, demonstrating the robustness of the inferred associations.

The BLASTp search against the nonredundant database showed a high degree of sequence similarity (up to 99%) between the query protein and other members of the YqeG family HAD IIIA‐type phosphatase protein (Table 3). As presented in Table 3, all of the chosen sequences had very low *E*‐values (between 1e‐123 and 3e‐123) and very high sequence identity (around 99.43%–100%), which shows that they are quite similar. Ten example sequences derived from the BLASTp findings endured MSA utilizing Jalview 2.11.1.3 (Figure 3). The alignment showed that the sequences were quite similar, especially in areas that were critical for their function. This suggests that the proteins have comparable structures and functions. Also, Jalview was used to create a phylogenetic tree based on the matched sequences (Figure 4). The sequences, mostly from *S. aureus*, grouped together in clusters that were quite similar to each other, showing how they evolved. The tight clustering pattern matches the high sequence identity seen in Table 3, which supports the idea that these proteins are very similar and probably do the same biological tasks. The combined MSA and phylogenetic studies indicate that YqeG family HAD IIIA‐type phosphatases are conserved, providing insights into their evolutionary trajectory across various bacterial species.

**Table 3:** Results of the BLASTp search revealing significant alignments among HP OHS92176.1 and other YqeG family HAD IIIA‐type phosphatases.

Protein description	Organism	Length (aa)	*E*‐value	Identity (%)	Accession number
MULTISPECIES: YqeG family HAD IIIA‐type phosphatase	*Staphylococcus* spp.	356	1e‐123	100.00%	WP_000524903
YqeG family HAD IIIA‐type phosphatase	*Staphylococcus aureus*	355	2e‐123	99.43%	EJX2069322
		355	2e‐123	99.43%	EOB7661906
		355	2e‐123	99.43%	HDB3207721
		355	2e‐123	99.43%	WP_033856751
		355	2e‐123	99.43%	WP_310651392
		355	2e‐123	99.43%	WP_182078268
		355	3e‐123	99.43%	HEH1175399
		355	3e‐123	99.43%	WP_423145876
		355	3e‐123	99.43%	HDG4350403

### 3.5. Analysis of 2D Structure

**Figure 5:**
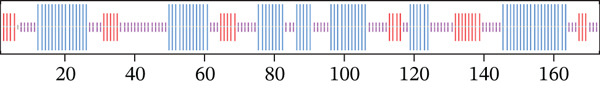
The 2D structure of HP along the amino acid sequence predicted by using the SOPMA tool. The diagram depicts *α*‐helices as blue bars, *β*‐strands as red bars, and random coils as magenta dotted lines. Each color pattern signifies the expected configuration of particular residues within the sequence. The *x*‐axis signifies the positions of amino acid residues, enabling the observation of the sequential arrangement and periodicity of structural motifs. The existence of alternate *α*‐helical and *β*‐strand regions, interspersed with random coil segments, signifies that the protein adopts a mixed *α*/*β* conformation, a characteristic commonly associated with globular proteins exhibiting compact and stable tertiary structures.

**Figure 6:**
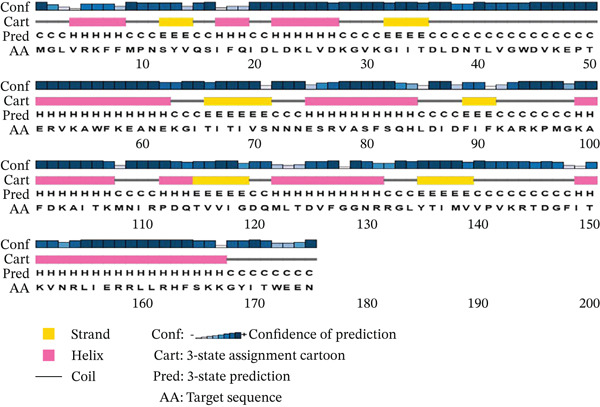
The 2D structure model of the targeted HP by the PSIPRED software. This visual depiction of the PSIPRED service is composed of four distinct elements. In the initial section, the bars vary in height. The confidence score correlates directly with the height of the bar. In the second part, the *α*‐helix is represented in pink, the *β*‐sheets or strands are shown in yellow, and the coils are depicted in gray. A coil connects a particular beta sheet to a particular *α*‐helix. The third section illustrates the secondary structure of a protein using an alphabetical representation; the letters H, C, and E correspond to *α*‐helix, coils, and *β*‐sheets or extended strands, respectively. The final section presents the AAs arranged in alphabetical order.

The 2D structure of the HP was designated utilizing SOPMA software together with the PSIPRED tool (Figures 5 and 6). The SOPMA tool estimates showed that the *α*‐helix was the most common structural element, making up 48% of the structure. This result was followed by the random coil at 24.57%, the extended strand at 17.71%, and the beta turn at 9.71%. In contrast, PSIPRED predictions revealed a slightly distinct distribution, indicating a random coil of 31.11% as the most abundant component, followed by an *α*‐helix of 40% and an extended strand of 28.89%.

### 3.6. 3D Structure Prediction and Quality Assessment

**Figure 7:**
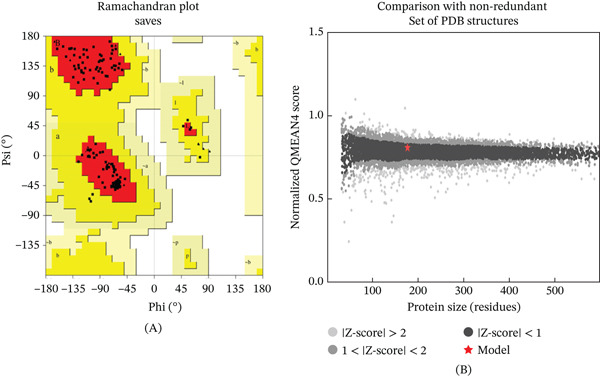
Quality evaluation of the model HP OHS92176.1 tertiary structure. (A) The PROCHECK program verified the Ramachandran plot of the modeled structure. (B) The graphical representation of the model′s QMEAN score demonstrates a significant agreement between the predicted structure and experimentally determined structures of comparable size, with a QMEAN4 value of −0.72.

**Figure 8:**
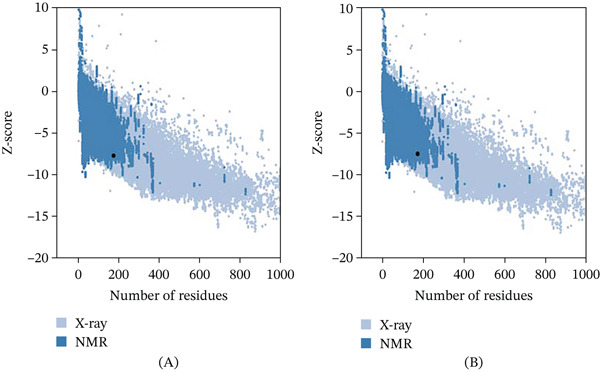
The z‐scores of the assessed HP OHS92176.1 and template protein calculated by using the ProSA‐web software. Here, (A) represents the structure of model HP, with a z‐score of −7.74, whereas (B) denotes the template proteins, which have a z‐score of −7.44. The positions of the two structures align with those commonly observed in natural proteins of comparable size that have been established through experimental methods such as NMR and X‐ray.

With the use of the template A0A2C6WKT6.1.A, the tertiary structure of the HP was retrieved from the SWISS‐MODEL program. This structure exhibited a sequence similarity of 71.43% with the target protein. We used PROCHECK, QMEAN, Verify3D, ERRAT, and ProSA‐web server to evaluate our 3D structural simulation of HP. The analysis of the Ramachandran plot (Figure 7A) revealed that 5.1% of residues occupied the additionally permissible regions, while 94.9% of residues are situated in the most favorable areas (Table 4). No residues were detected in the permitted or prohibited regions [49]. The findings indicate that most AAs exhibit phi‐psi angles typical of a right‐handed *α*‐helix, demonstrating the structural stability and flexibility of the HP. The QMEAN server predicted a QMEAN4 value of 0.72 and a |z‐score| of less than 1. This indicates that the model scores are consistent with what would be expected from a similarly sized structure that was determined experimentally (Figure 7B). The ERRAT study produced a quality factor of 98.788, indicating that the structure of protein is expected to be of high quality. The z‐score of a model acts as a measure of its overall quality and is utilized to evaluate if the input structure lies within the standard range reported for native proteins of comparable size. The z‐score of −7.74 for the tested HP (Figure 8A) and −7.44 for the template protein (Figure 8B) suggests that the model HP falls within the standard range generally observed for natural proteins of comparable sizes [50]. These assessments not only validate the accuracy of the structural predictions but also enhance confidence in the anticipated biological functionality of the HP.

**Table 4:** The Ramachandran plot statistical analysis of the 3D model for the HP OHS92176.1.

Parameters of Ramachandran plot statistics	Number of AA residues	Percentage (%)
Residues in the most favored locations [A, B, L]	148	94.9
Additional permitted areas for residues [a, b, l, p]	8	5.1
Residues in areas that are liberally permitted [~a, ~b, ~l, ~p]	0	0.0
Residues in prohibited areas	0	0.0
Number of residues that are not glycine (Gly) or proline (Pro)	156	100.0
Number of termination residues (excluding both Gly and Pro)	2	—
Number of Gly residues (represented as triangles)	12	—
Number of Pro residues	5	—
Total number of AA residues	175	—

### 3.7. Analysis of PPIs

**Figure 9:**
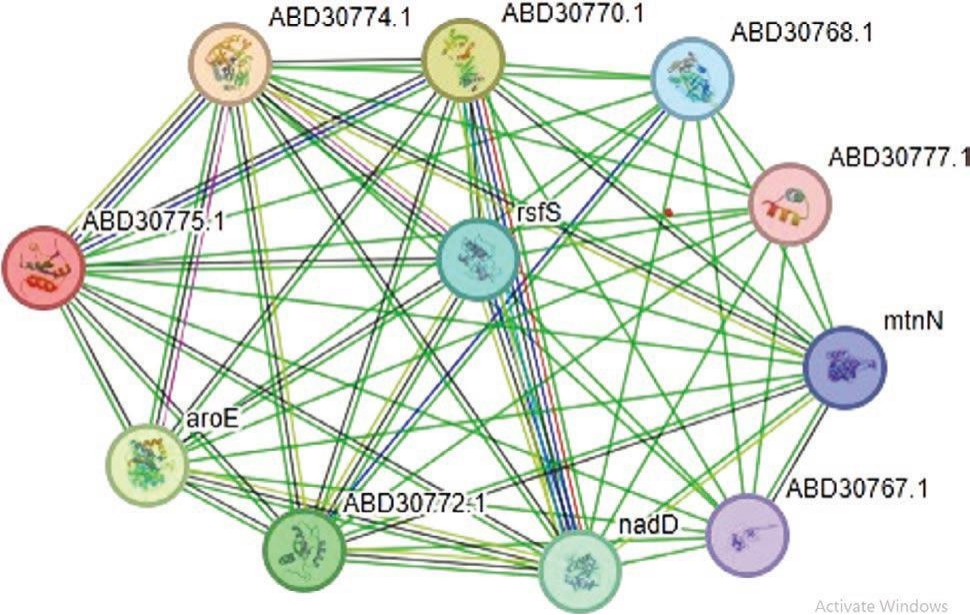
The network of protein–protein interactions analysis of the studied HP OHS92176.1 obtained by the STRING database version 12. Proteins are represented as nodes, whereas interactions between two proteins are represented by edges. Here, in this STRING analysis, ABD30775.1 is the annotated HP OHS92176.1. ABD30774.1, ABD30770.1, and ABD30772.1 are conserved hypothetical proteins. aroE, nadD, rsfS, and mtnN denote proteins involved in aromatic AA biosynthesis, NAD biosynthesis, ribosomal silencing, and methionine salvage pathway, respectively.

The network of PPIs was developed using the STRING database to investigate the functional relationship of the HP. The specific HP (OHS92176.1) corresponds to the STRING database′s accession number ABD30775.1, as various databases can provide the same alternative identifiers for proteins. The putative protein, ABD30775.1, is functionally linked to several other proteins, including ABD30774.1, ABD30770.1, ABD30768.1, ABD30777.1, mtnN, rsfS, ABD30767.1, nadD, ABD30772.1, and aroE, as illustrated in Figure 9 [51]. Numerous conserved hypothetical proteins (such as ABD30774.1, ABD30770.1, and ABD30772.1) and functionally characterized proteins, including aroE (involved in aromatic AA biosynthesis), nadD (NAD biosynthesis), rsfS (ribosomal silencing), and mtnN (methionine salvage pathway), exhibited a strong association with it. The significant interconnectedness and elevated interaction scores (reaching 0.910) suggest that the target protein plays a crucial role in essential cellular pathways. The findings indicate that the protein could be involved in significant metabolic processes and contribute to the cellular stability of *S. aureus*. Additional exploration of its particular roles and interactions may clarify its impact on cellular balance and the overall physiology of the organism.

### 3.8. Detection of Active Site

**Figure 10:**
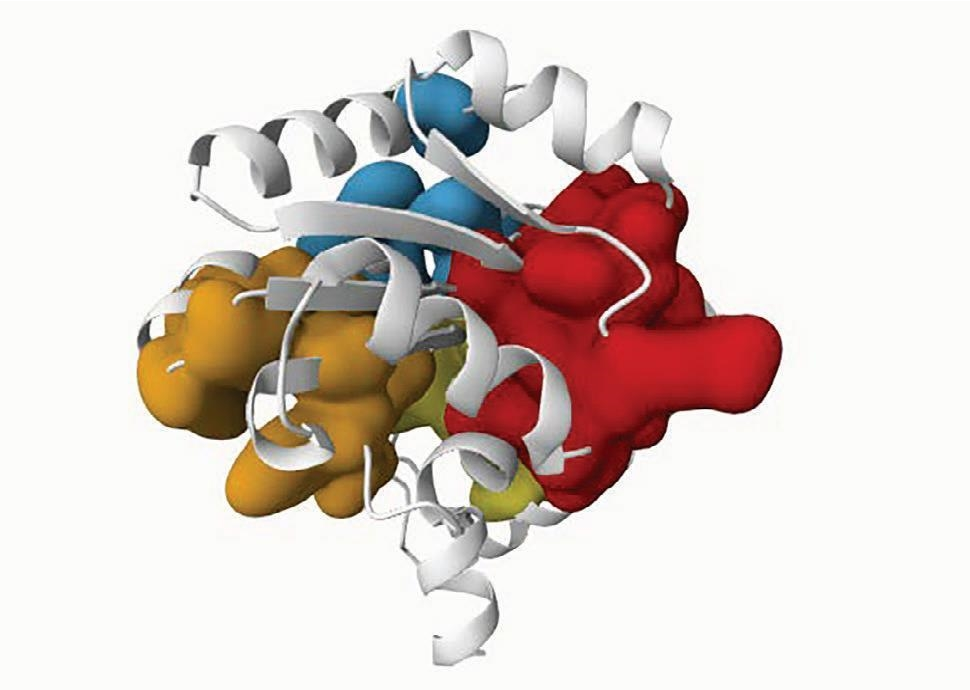
The top‐ranked active site within the three‐dimensional configurations of HP OHS92176.1 predicted by PrankWeb.

The PrankWeb software had been employed to detect active sites inside the 3D structure, uncovering four pockets ranked by score. Rank 1 (Red) received a score of 23.75, with a probability of 0.875 and involving 19 residues. Rank 2 (Yellow) has a score of 5.54, indicating a probability of 0.279 and comprising 14 residues. Rank 3 (Brown) is scored at 4.42, with a probability of 0.199 and containing 13 residues. Rank 4 (Blue) shows a score of 1.67, with a probability of 0.027 and including 8 residues. The rankings indicate a substantial differential in the stability or interaction potential among the pockets, with Rank 1 demonstrating a markedly greater probability and score (Figure 10).

### 3.9. MD Analysis

MD analysis was conducted through AutoDock Vina to determine the binding affinity between the modeled HP and six chosen ligands: quercetin, kaempferol, naringenin, apigenin, catechin, and curcumin. Kaempferol had the greatest binding affinity of all the ligands examined (−8.3 kcal/mol), followed by apigenin and naringenin (−8.2 kcal/mol each). Quercetin and catechin exhibited robust interactions with binding energies of −8.1 kcal/mol, whereas curcumin had a little lower although still advantageous binding energy of −7.9 kcal/mol. The docking results indicated a limited range of binding energies (−7.9 to −8.3 kcal/mol) for all ligands, implying consistent ligand–protein interactions. (Figure 11).

Figure 11The docking analysis of the six ligands in the active site of the HP by AutoDock Vina. (A) Quercetin‐bound HP, (C) kaempferol‐bound HP, (E) naringenin‐bound HP, (G) apigenin‐bound HP, (I) catechin‐bound HP, and (K) curcumin‐bound HP (analyzed by PyMOL); (B) Crucial interacting residues of the HP with Quercetin, (D) crucial interacting residues of the HP with kaempferol, (F) crucial interacting residues of the HP with naringenin, (H) crucial interacting residues of the HP with apigenin, (J) crucial interacting residues of the HP with catechin, and (L) crucial interacting residues of the HP with curcumin (depicted using BIOVIA Discovery Studio visualizer).

**Figure:**
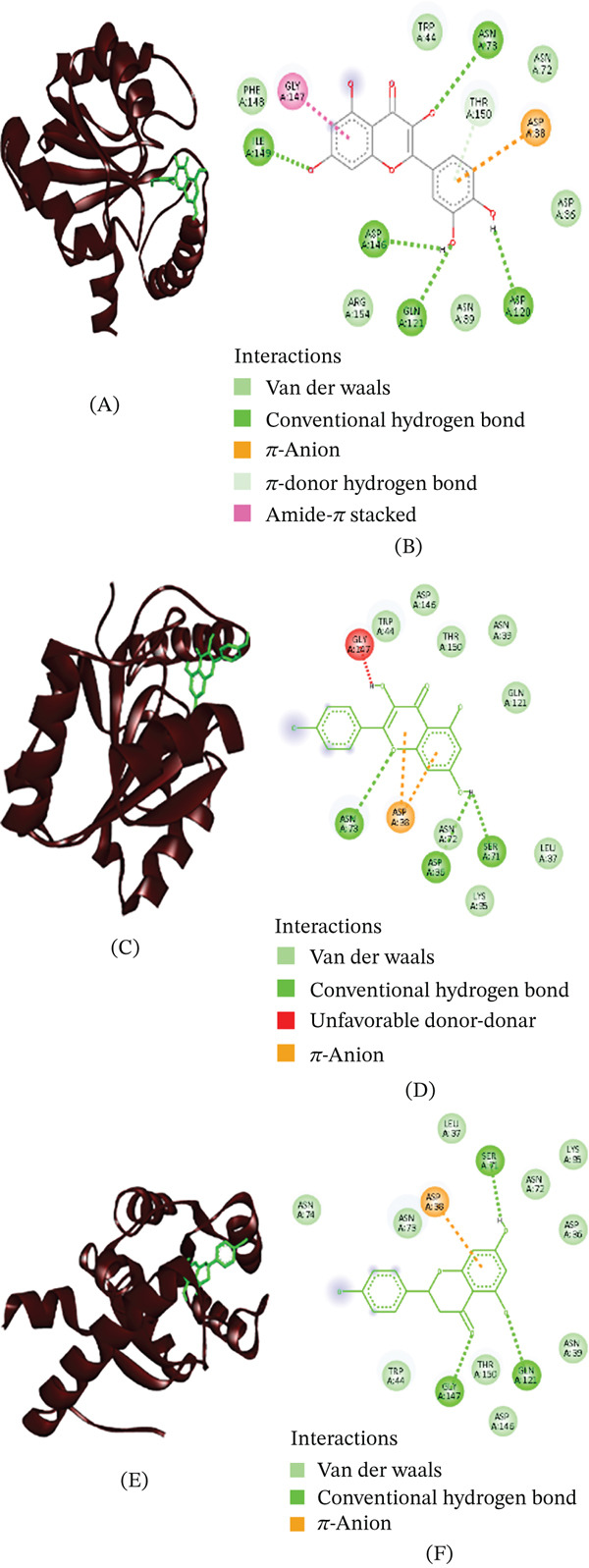
(A)

**Figure:**
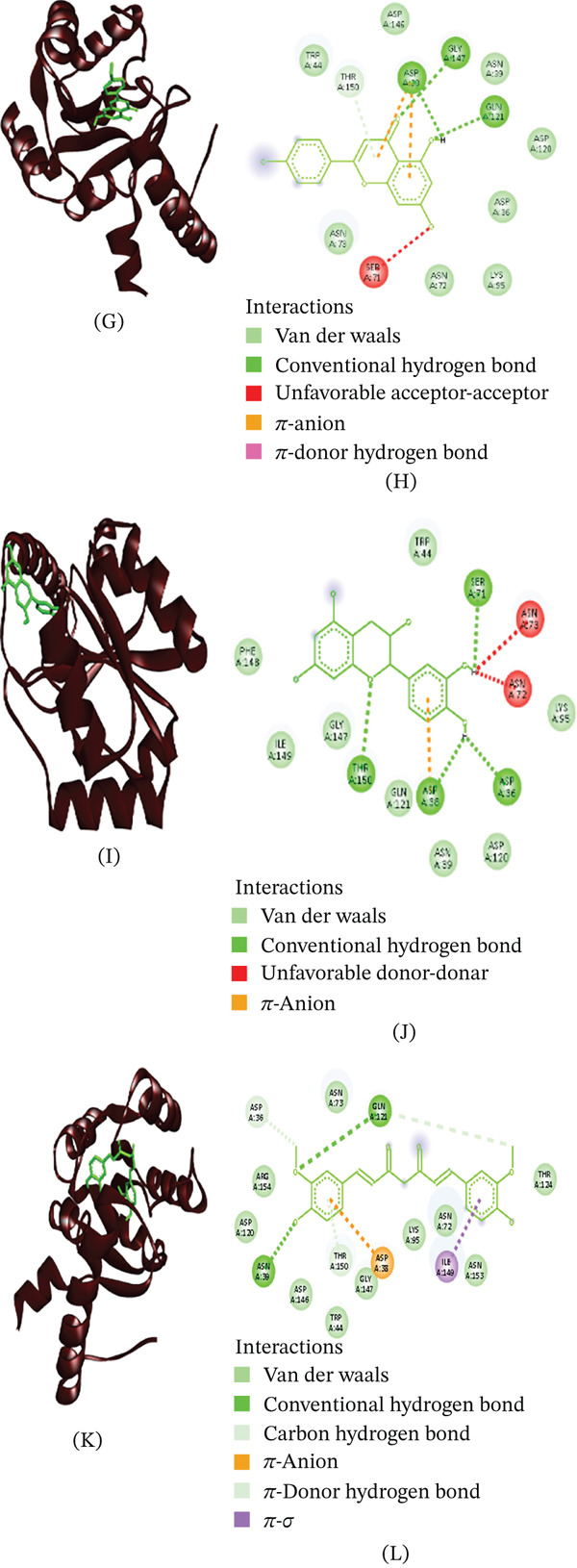
(B)

## 4. Discussion

The current study was aimed at in silico annotation of a hypothetical protein (OHS92176.1) of *S. aureus* strain HMSC77AO5, which was not characterized earlier. The study led to the classification of the HP in the YqeG family of the HAD IIIA‐type phosphatase superfamily. In this work, we integrated physicochemical profiling, conserved domain identification, structural modeling, evolutionary analysis, PPI mapping, and MD to build a coherent functional and structural framework of the HP and to demonstrate its biological and therapeutic relevance. The physicochemical attributes of the annotated HP strongly endorsed its identity as a functional intracellular enzyme. The estimated MW (~20 kDa) was within the range of YqeG family members, and the basic isoelectric point (pI 9.73) indicated an enrichment of positively charged residues that facilitate binding with the negatively charged phosphate groups during catalysis [52, 53]. Such electrostatic interactions are critical for phosphatase activity [54]. A negative GRAVY value denoted hydrophilicity, consistent with cytoplasmic localization, and the low II suggested protein stability. These properties are common for metabolically active phosphatase enzymes [55, 56]. Conserved domain analysis provided persuasive evidence for the functional annotation, consistently identifying the HP as a HAD IIIA‐type phosphatase, with a conserved domain encompassing residues 6–168. The very low *E*‐value (1.48e‐86) provides strong statistical support for this functional alignment, which shows that the HP is in that enzyme group. Enzymes of the HAD superfamily are known to catalyze diverse dephosphorylation and hydrolytic reactions, playing essential roles in central metabolism, detoxification pathways, and cellular signaling [5, 57]. YqeG homologs have been implicated in nucleotide metabolism and bacterial colony formation [58], suggesting that the studied protein may contribute to similar physiological processes in *S. aureus*. The robustness of this annotation is further strengthened by concordant results across multiple databases (NCBI‐CDD, Pfam, and InterProScan). Evolutionary analysis showed a high degree of sequence conservation (~99% sequence identity) among YqeG family members from different strains of *S. aureus* [2]. This remarkable level of conservation typically demonstrates robust selective strain to maintain function, indicating that the protein likely plays an essential biological role for the survival of bacteria [59]. This conservation reduces the likelihood of the protein being functionally redundant or specific to a particular strain, thereby reinforcing its categorization as a conserved enzymatic element within bacterial metabolic networks. Secondary structure analysis predicted a predominance of *α*‐helices (44%–48% of the structure) along with *β*‐strands and random coils, forming a canonical *α*/*β* fold characteristic of HAD superfamily enzymes [5]. This structural architecture has to be known to support a conserved catalytic core, often stabilized by surrounding helices that facilitate substrate binding and enzymatic activity [60]. The reliability of this structural arrangement was further confirmed by homology‐based tertiary structure modeling. Despite moderate template identity (71.43%), the model exhibited excellent stereochemical quality, with 94.9% of residues in favored regions of the Ramachandran plot and none in disallowed regions. In addition, complementary validation metrics, such as QMEAN, ERRAT, and ProSA‐web scores, fell within acceptable ranges for experimentally resolved protein structures, emphasizing the structural robustness and biological plausibility of the predicted model HP [40, 41, 61]. Functional context was further elucidated through PPI network analysis, which revealed strong associations of the HP with proteins involved in essential metabolic pathways, including nicotinamide adenine dinucleotide (NAD) biosynthesis (nadD), aromatic AA biosynthesis (aroE), and ribosomal regulation (rsfS). The high‐confidence interaction scores (up to 0.910) show that the protein functions within an interconnected metabolic framework rather than as an isolated entity [62]. Its association with key biosynthetic and regulatory proteins underscores its potential contribution to cellular homeostasis and bacterial viability, further reinforcing its biological significance.

MD analysis was subsequently employed to assess ligand binding interactions based on the identification of a high‐confidence active site [63]. The selected naturally occurring polyphenolic compounds, quercetin, kaempferol, naringenin, apigenin, catechin, and curcumin, exhibited consistent and favorable binding in the predicted active site with binding energies ranging from −7.9 to −8.3 kcal/mol. Among these, kaempferol was the best binder, with three hydrogen bonds with residues Asp36, Ser71, and Asn73. The relatively narrow energy distribution among structurally diverse ligands suggests that the active site is flexible enough to accommodate multiple classes of inhibitors [44–48]. The docking result highlights the investigated HP as a promising target for antibacterial drug development.

Despite these promising findings, it is important to acknowledge the limitations inherent in computational studies. Functional predictions derived from sequence and structural homology, whereas robust, require experimental validation. Biochemical assays, site‐directed mutagenesis, and in vitro/in vivo functional studies are essential to confirm enzymatic activity and biological role. Furthermore, although MD provides valuable insights into ligand binding, it does not fully capture protein dynamics. Therefore, extended molecular dynamics (MD) simulations are recommended to evaluate the stability and conformational behavior of protein–ligand complexes under physiological conditions.

The present study uncovers the classification of the protein as a conserved YqeG family HAD IIIA‐type phosphatase involved in vital metabolic processes in *S. aureus*. The structural stability, evolutionary conservation, and ligand‐binding potential of this HP highlight its promise as a target for antibiotic drug development and suggest the need for further experimental validation.

## 5. Conclusion

In the present study, several bioinformatics tools were used to systematically characterize a hypothetical protein encoded by *S. aureus* providing strong computational insights into its structural identity, functional annotation, and therapeutic potential. Sequence analysis based on domains indicated that the protein belongs to the YqeG family of HAD superfamily IIIA‐type phosphatases that play significant roles in phosphoryl transfer and metabolic regulatory reactions. This classification is further supported by approximately 99% sequence identity among YqeG homologs across various *S. aureus* strains, indicating significant functional conservation. Physicochemical characterization confirmed that the protein possessed properties consistent with a stable, soluble intracellular enzyme. The homology‐based structural modeling was evaluated by different stereochemical quality assessment methods, confirming its reliability and suitability for downstream in silico investigations. Analysis of the PPI network indicated that the protein is functionally linked to critical cellular phosphorylation and metabolic regulatory processes vital for the survival and homeostasis of *S. aureus*. MD studies involving various antibacterial agents suggested that the protein may serve as a druggable target for the development of novel anti‐*S. aureus* therapeutic agents. However, the predicted cytoplasmic location of the protein limits its application as a surface‐exposed vaccine candidate. The results of this work provide a robust computational framework for future structure‐based drug discovery efforts to develop *S. aureus* YqeG‐family HAD phosphatases as novel therapeutic agents. This study was performed exclusively using computational predictions; thus, it is suggested to experimentally validate the protein′s inhibitory susceptibility and biological activity through in vitro and in vivo examinations. The findings of this study facilitate subsequent computational and experimental inquiries into proteins in bacteria whose functions remain inadequately characterized.

**Nomenclature**
HP: hypothetical protein
MW: molecular weight
HAD: haloacid dehalogenase
BLAST: Basic Local Alignment Search Tool
MSA: multiple‐sequence alignment
QMEAN: qualitative model energy analysis
ProSA: protein structure analysis
pI: isoelectric point
AI: aliphatic index
3D: three‐dimensional
GMQE: Global Model Quality Estimation
2D: two‐dimensional
II: instability index
PSIPRED: PSI‐Blast Based Secondary Structure PREDiction
SOPMA: Self‐Optimized Prediction Method with Alignment
MD: molecular docking

## Author Contributions

**Mehedi Hasan Chowdhury:** writing—original draft, methodology, visualization, verification, computational resources, investigation, formal analysis, data collection, conceptualization. **Md. Reaz Morshed:** writing—review and editing, methodology, graphing, software, verification, data collection, validation, supervision. **Mohammad Jane Alam:** review, formal editing, writing—original draft. **Sazzad Hossain:** formal analysis, data interpretation, manuscript writing. **Hasna Hena:** intellectual input throughout the study.

## Funding

No funding was received for this manuscript.

## Conflicts of Interest

The authors declare no conflicts of interest.

## Data Availability

The data that support the findings of this study are available on request from the corresponding author. The data are not publicly available due to privacy or ethical restrictions.

## References

[1] Varma P. B. S. , Adimulam Y. B. , and Kodukula S. , In Silico Functional Annotation of a Hypothetical Protein From Staphylococcus aureus, Journal of Infection and Public Health. (2015) 8, no. 6, 526–532, 10.1016/j.jiph.2015.03.007, 26025048. 26025048

[2] Galperin M. Y. and Koonin E. V. , ‘Conserved hypothetical’ Proteins: Prioritization of Targets for Experimental Study, Nucleic Acids Research. (2004) 32, no. 18, 5452–5463, 10.1093/nar/gkh885, 15479782. 15479782 PMC524295

[3] Naveed M. , Makhdoom S. I. , Abbas G. , Safdari M. , Farhadi A. , Habtemariam S. , Shabbir M. A. , Jabeen K. , Asif M. F. , and Tehreem S. , The Virulent Hypothetical Proteins: The Potential Drug Target Involved in Bacterial Pathogenesis, Mini Reviews in Medicinal Chemistry. (2022) 22, no. 20, 2608–2623, 10.2174/1389557522666220413102107. 35422211

[4] Allen K. and Dunaway-Mariano D. , Phosphoryl Group Transfer: Evolution of a Catalytic Scaffold, Trends in Biochemical Sciences. (2004) 29, no. 9, 495–503, 10.1016/j.tibs.2004.07.008, 15337123. 15337123

[5] Burroughs A. M. , Allen K. N. , Dunaway-Mariano D. , and Aravind L. , Evolutionary Genomics of the HAD Superfamily: Understanding the Structural Adaptations and Catalytic Diversity in a Superfamily of Phosphoesterases and Allied Enzymes, Journal of Molecular Biology. (2006) 361, no. 5, 1003–1034, 10.1016/j.jmb.2006.06.049, 16889794. 16889794

[6] Pathira Kankanamge L. S. , Ruffner L. A. , Touch M. M. , Pina M. , Beuning P. J. , and Ondrechen M. J. , Functional Annotation of Haloacid Dehalogenase Superfamily Structural Genomics Proteins, Biochemical Journal. (2023) 480, no. 19, 1553–1569, 10.1042/BCJ20230057, 37747786. 37747786

[7] Fayisa W. O. and Tuli N. F. , Review on Staphylococcus aureus, International Journal of Nursing and Health Care Research. (2020) 1, 1–8.

[8] Payne D. J. , Gwynn M. N. , Holmes D. J. , and Pompliano D. L. , Drugs for Bad Bugs: Confronting the Challenges of Antibacterial Discovery, Nature Reviews Drug Discovery. (2007) 6, no. 1, 29–40, 10.1038/nrd2201, 17159923. 17159923

[9] Abebe A. A. and Birhanu A. G. , Methicillin-Resistant Staphylococcus aureus: Molecular Mechanisms Underlying Drug Resistance Development and Novel Strategies to Combat, Infection and Drug Resistance. (2023) 16, 7641–7662, 10.2147/IDR.S428103, 38111667. 38111667 PMC10726795

[10] Marklevitz J. and Harris L. K. , Improved Annotations of 23 Differentially Expressed Hypothetical Proteins in Methicillin Resistant S.aureus, Bioinformation. (2017) 13, no. 4, 104–110, 10.6026/97320630013104, 28539731. 28539731 PMC5429968

[11] School K. , Marklevitz J. , Schram W. K. , and Harris L. K. , Predictive Characterization of Hypothetical Proteins in Staphylococcus aureus NCTC 8325, Bioinformation. (2016) 12, no. 3, 209–220, 10.6026/97320630012209, 28149057. 28149057 PMC5267966

[12] Rabbi M. F. , Akter S. A. , Hasan M. J. , and Amin A. , In Silico Characterization of a Hypothetical Protein From Shigella dysenteriae ATCC 12039 Reveals a Pathogenesis-Related Protein of the Type-VI Secretion System, Bioinformatics and Biology Insights. (2021) 15, 11779322211011140, 10.1177/11779322211011140, 33994781. 33994781 PMC8076777

[13] Mohan R. and Venugopal S. , Computational Structural and Functional Analysis of Hypothetical Proteins of Staphylococcus aureus, Bioinformation. (2012) 8, no. 15, 722–728, 10.6026/97320630008722, 23055618. 23055618 PMC3449381

[14] Jumper J. , Evans R. , Pritzel A. , Green T. , Figurnov M. , Ronneberger O. , Tunyasuvunakool K. , Bates R. , Žídek A. , Potapenko A. , Bridgland A. , Meyer C. , Kohl S. A. A. , Ballard A. J. , Cowie A. , Romera-Paredes B. , Nikolov S. , Jain R. , Adler J. , Back T. , Petersen S. , Reiman D. , Clancy E. , Zielinski M. , Steinegger M. , Pacholska M. , Berghammer T. , Bodenstein S. , Silver D. , Vinyals O. , Senior A. W. , Kavukcuoglu K. , Kohli P. , and Hassabis D. , Highly Accurate Protein Structure Prediction With AlphaFold, Nature. (2021) 596, no. 7873, 583–589, 10.1038/s41586-021-03819-2. 34265844 PMC8371605

[15] Prava J. , Pranavathiyani G. , and Pan A. , Functional Assignment for Essential Hypothetical Proteins of Staphylococcus aureus N315, International Journal of Biological Macromolecules.(2018) 108, 765–774, 10.1016/j.ijbiomac.2017.10.169, 29111265. 29111265

[16] Bin H. A. , Sappati S. , Krzemieniecki R. , Worobo R. , and Szweda P. , In Silico Functional Annotation and Structural Characterization of Hypothetical Proteins in Bacillus paralicheniformis and Bacillus subtilis Isolated from Honey, ACS Omega. (2025) 10, no. 9, 8993–9006. 40092810 10.1021/acsomega.4c07105 PMC11904672

[17] Lionta E. , Spyrou G. , Vassilatis K. , and Cournia Z. , Structure-Based Virtual Screening for Drug Discovery: Principles, Applications and Recent Advances, Current Topics in Medicinal Chemistry. (2014) 14, no. 16, 1923–1938, 10.2174/1568026614666140929124445, 25262799. 25262799 PMC4443793

[18] Mirzaei B. , Babaei R. , Zeighami H. , Dadar M. , and Soltani A. , Staphylococcus aureus Putative Vaccines Based on the Virulence Factors: A Mini-Review, Frontiers in Microbiology. (2021) 12, 704247, 10.3389/fmicb.2021.704247, 34539603. 34539603 PMC8447878

[19] Benson D. A. , Karsch-Mizrachi I. , Lipman D. J. , and Wheeler D. L. , An inbred 129SvEv GFPCre transgenic mouse that deletes loxP‐flanked genes in all tissues, Nucleic Acids Research. (2003) 31, no. 1, 23–27, 10.1093/nar/gkg057, 12519940. 12736323 PMC156060

[20] Gasteiger E. , Hoogland C. , Gattiker A. , Duvaud S. , Wilkins M. , Appel R. D. , and Bairoch A. , Protein Identification and Analysis Tools on the ExPASy Server, The Proteomics Protocols Handbook, 2005, Humana pres, 10.1385/1-59259-890-0:571.

[21] Yu C. S. , Chen Y. C. , Lu C. H. , and Hwang J. K. , Prediction of Protein Subcellular localization, Function and Bioinformatics. (2006) 64, no. 3, 643–651, 10.1002/prot.21018. 16752418

[22] Shen H. B. and Chou K. C. , Gneg-mPLoc: A Top-Down Strategy to Enhance the Quality of Predicting Subcellular Localization of Gram-Negative Bacterial Proteins, Journal of Theoretical Biology. (2010) 264, no. 2, 326–333, 10.1016/j.jtbi.2010.01.018, 20093124. 20093124

[23] Hebditch M. , Carballo-Amador M. A. , Charonis S. , Curtis R. , and Warwicker J. , Protein–Sol: A Web Tool for Predicting Protein Solubility From Sequence, Bioinformatics. (2017) 33, no. 19, 3098–3100, 10.1093/bioinformatics/btx345, 28575391. 28575391 PMC5870856

[24] Lu S. , Wang J. , Chitsaz F. , Derbyshire M. K. , Geer R. C. , Gonzales N. R. , Gwadz M. , Hurwitz D. I. , Marchler G. H. , Song J. S. , Thanki N. , Yamashita R. A. , Yang M. , Zhang D. , Zheng C. , Lanczycki C. J. , and Marchler-Bauer A. , CDD/SPARCLE: The Conserved Domain Database in 2020, Nucleic Acids Research. (2020) 48, no. D1, D265–D268, 10.1093/nar/gkz991, 31777944. 31777944 PMC6943070

[25] Jones P. , Binns D. , Chang H. Y. , Fraser M. , Li W. , McAnulla C. , McWilliam H. , Maslen J. , Mitchell A. , Nuka G. , Pesseat S. , Quinn A. F. , Sangrador-Vegas A. , Scheremetjew M. , Yong S. Y. , Lopez R. , and Hunter S. , InterProScan 5: Genome-Scale Protein Function Classification, Bioinformatics. (2014) 30, no. 9, 1236–1240, 10.1093/bioinformatics/btu031, 24451626. 24451626 PMC3998142

[26] Altschul S. F. , Gish W. , Miller W. , Myers E. W. , and Lipman D. J. , Basic Local Alignment Search Tool, Journal of Molecular Biology. (1990) 215, no. 3, 403–410, 10.1016/S0022-2836(05)80360-2. 2231712

[27] Waterhouse A. M. , Procter J. B. , Martin D. M. , Clamp M. , and Barton G. J. , Jalview Version 2—A Multiple Sequence Alignment Editor and Analysis Workbench, Bioinformatics. (2009) 25, no. 9, 1189–1191, 10.1093/bioinformatics/btp033, 19151095. 19151095 PMC2672624

[28] Saitou N. and Nei M. , The Neighbor-Joining Method: A New Method for Reconstructing Phylogenetic Trees, Molecular Biology and Evolution. (1987) 4, no. 4, 406–425, 3447015. 3447015 10.1093/oxfordjournals.molbev.a040454

[29] Felsenstein J. , Confidence Limits on Phylogenies: An Approach Using the Bootstrap, Evolution. (1985) 39, no. 4, 783–791, 10.1111/j.1558-5646.1985.tb00420.x, 28561359. 28561359

[30] Buchan D. W. A. , Moffat L. , Lau A. , Kandathil S. M. , and Jones D. T. , Deep Learning for the PSIPRED Protein Analysis Workbench, Nucleic Acids Research. (2024) 52, no. W1, W287–W293, 10.1093/nar/gkae328, 38747351. 38747351 PMC11223827

[31] Geourjon C. and Deléage G. , SOPMA: Significant Improvements in Protein Secondary Structure Prediction by Consensus Prediction From Multiple Alignments, Bioinformatics. (1995) 11, no. 6, 681–684, 10.1093/bioinformatics/11.6.681, 8808585. 8808585

[32] Waterhouse A. , Bertoni M. , Bienert S. , Studer G. , Tauriello G. , Gumienny R. , Heer F. T. , de Beer T. A. P. , Rempfer C. , Bordoli L. , Lepore R. , and Schwede T. , SWISS-MODEL: Homology Modelling of Protein Structures and Complexes, Nucleic Acids Research. (2018) 46, no. W1, W296–W303, 10.1093/nar/gky427, 29788355. 29788355 PMC6030848

[33] Laskowski R. A. , MacArthur M. W. , Moss D. S. , and Thornton J. M. , PROCHECK: A Program to Check the Stereochemical Quality of Protein Structures, Journal of Applied Crystallography. (1993) 26, no. 2, 283–291, 10.1107/S0021889892009944.

[34] Eisenberg D. , Lüthy R. , and Bowie J. U. , [20] VERIFY3D: Assessment of Protein Models With Three-Dimensional Profiles, Methods in Enzymology. (1997) 277, 396–404, 10.1016/S0076-6879(97)77022-8. 9379925

[35] Colovos C. and Yeates T. O. , Verification of Protein Structures: Patterns of Nonbonded Atomic Interactions, Protein Science. (1993) 2, no. 9, 1511–1519, 10.1002/pro.5560020916, 8401235. 8401235 PMC2142462

[36] Benkert P. , Biasini M. , and Schwede T. , Toward the Estimation of the Absolute Quality of Individual Protein Structure Models, Bioinformatics. (2011) 27, no. 3, 343–350, 10.1093/bioinformatics/btq662. 21134891 PMC3031035

[37] Wiederstein M. and Sippl M. J. , ProSA-web: Interactive Web Service for the Recognition of Errors in Three-Dimensional Structures of Proteins, Nucleic Acids Research.(2007) 35, W407–W410, 10.1093/nar/gkm290, 17517781. 17517781 PMC1933241

[38] Szklarczyk D. , Gable A. L. , Nastou K. C. , Lyon D. , Kirsch R. , Pyysalo S. , Doncheva N. T. , Legeay M. , Fang T. , Bork P. , Jensen L. J. , and von Mering C. , The STRING Database in 2021: Customizable Protein–Protein Networks, and Functional Characterization of User-Uploaded Gene/Measurement Sets, Nucleic Acids Research. (2021) 49, no. D1, D605–D612, 10.1093/nar/gkaa1074, 33237311. 33237311 PMC7779004

[39] Jones S. and Thornton J. M. , Principles of Protein–Protein Interactions, Proceedings of the National Academy of Sciences. (1996) 93, no. 1, 13–20, 10.1073/pnas.93.1.13, 8552589. PMC40170 8552589

[40] Jakubec D. , Skoda P. , Krivak R. , Novotny M. , and Hoksza D. , PrankWeb 3: Accelerated Ligand-Binding Site Predictions for Experimental and Modelled Protein Structures, Nucleic Acids Research. (2022) W1, no. W1, W593–W597. 10.1093/nar/gkac389 PMC10353840 35609995

[41] Liang J. , Woodward C. , and Edelsbrunner H. , Anatomy of Protein Pockets and Cavities: Measurement of Binding Site Geometry and Implications for Ligand Design, Protein Science. (1998) 7, no. 9, 1884–1897, 10.1002/pro.5560070905, 9761470. 9761470 PMC2144175

[42] Trott O. and Olson A. J. , AutoDock Vina: Improving the Speed and Accuracy of Docking With a New Scoring function, efficient optimization, and multithreading, Journal of Computational Chemistry. (2010) 31, no. 2, 455–461, 10.1002/jcc.21334, 19499576. 19499576 PMC3041641

[43] Dos Santos R. N. , Ferreira L. G. , and Andricopulo A. D. , Practices in Molecular Docking and Structure-Based Virtual Screening, Computational Drug Discovery and Design, 2018, Springer, 31–50, 10.1007/978-1-4939-7756-7_3. 29594766

[44] Veiko A. G. , Olchowik-Grabarek E. , Sekowski S. , Roszkowska A. , Lapshina E. A. , Dobrzynska I. , Zamaraeva M. , and Zavodnik I. B. , Antimicrobial Activity of Quercetin, Naringenin and Catechin: Flavonoids Inhibit Staphylococcus aureus-Induced Hemolysis and Modify Membranes of Bacteria and Erythrocytes, Molecules. (2023) 28, no. 3, 10.3390/molecules28031252, 36770917. PMC9920354 36770917

[45] Wei X. , Wang W. , Hu R. , Gao X. , Li B. , Bai Y. , and Zhang J. , Advances in Kaempferol: Extraction, Biosynthesis, and Application with Antibacterial Agents, Antibiotics. (2025) 14, no. 12, 10.3390/antibiotics14121254, 41463756. PMC12729929 41463756

[46] Morimoto Y. , Baba T. , Sasaki T. , and Hiramatsu K. , Apigenin as an Anti-Quinolone-Resistance Antibiotic, International Journal of Antimicrobial Agents. (2015) 46, no. 6, 666–673, 10.1016/j.ijantimicag.2015.09.006, 26526895. 26526895

[47] Teow S. Y. , Liew K. , Ali S. A. , Khoo A. S. B. , and Peh S. C. , Antibacterial Action of Curcumin Against Staphylococcus aureus: A Brief Review, Journal of Tropical Medicine. (2016) 2016, 2853045, 10.1155/2016/2853045, 27956904. 27956904 PMC5124450

[48] Cushnie T. T. and Lamb A. J. , Antimicrobial Activity of Flavonoids, International Journal of Antimicrobial Agents. (2005) 26, no. 5, 343–356, 10.1016/j.ijantimicag.2005.09.002, 16323269. 16323269 PMC7127073

[49] Musso G. A. , Zhang Z. , and Emili A. , Experimental and Computational Procedures for the Assessment of Protein Complexes on a Genome-Wide Scale, Chemical Reviews. (2007) 107, no. 8, 3585–3600, 10.1021/cr0682857, 17630806. 17630806

[50] Binbay F. A. , Rathod D. C. , George A. A. P. , and Imhof D. , Quality Assessment of Selected Protein Structures Derived From Homology Modeling and AlphaFold, Pharmaceuticals. (2023) 16, no. 12, 10.3390/ph16121662, 38139789. PMC10747200 38139789

[51] Franceschini A. , Szklarczyk D. , Frankild S. , Kuhn M. , Simonovic M. , Roth A. , Lin J. , Minguez P. , Bork P. , Von Mering C. , and Jensen L. J. , STRING v9.1: Protein–Protein Interaction Networks, With Increased Coverage and Integration, Nucleic Acids Research. (2013) 41, no. D1, D808–D815. 23203871 10.1093/nar/gks1094 PMC3531103

[52] Terán-Ramírez C. , Mares-Alejandre R. E. , Estrada-González A. L. , Muñoz-Muñoz P. L. , and Ramos-Ibarra M. A. , Structure-Function Relationship Study of a Secretory Amoebic Phosphatase: A Computational-Experimental Approach, International Journal of Molecular Sciences. (2021) 22, no. 4, 10.3390/ijms22042164, 33671604. PMC7926622 33671604

[53] Ning J. , Sala M. , Reina J. , Kalagiri R. , Hunter T. , and McCullough B. S. , Histidine Phosphorylation: Protein Kinases and Phosphatases, International Journal of Molecular Sciences. (2024) 25, no. 14, 10.3390/ijms25147975, 39063217. PMC11277029 39063217

[54] Tian C. , Yang J. , Liu C. , Chen P. , Zhang T. , Men Y. , Ma H. , Sun Y. , and Ma Y. , Engineering Substrate Specificity of HAD Phosphatases and Multienzyme Systems Development for the Thermodynamic-Driven Manufacturing Sugars, Nature Communications. (2022) 13, no. 1, 10.1038/s41467-022-31371-8, 35739124. PMC9226320 35739124

[55] Barrozo A. , Duarte F. , Bauer P. , Carvalho A. T. , and Kamerlin S. C. , Cooperative Electrostatic Interactions Drive Functional Evolution in the Alkaline Phosphatase Superfamily, Journal of the American Chemical Society. (2015) 137, no. 28, 9061–9076, 10.1021/jacs.5b03945, 26091851. 26091851 PMC4513756

[56] Saavedra D. E. M. and Baltar F. , Multifunctionality of Alkaline Phosphatase in Ecology and Biotechnology, Current Opinion in Biotechnology. (2025) 91, 103229, 10.1016/j.copbio.2024.103229, 39615073. 39615073

[57] Frickey T. and Lupas A. N. , Phylogenetic Analysis of AAA Proteins, Journal of Structural Biology. (2004) 146, no. 1-2, 2–10, 10.1016/j.jsb.2003.11.020. 15037233

[58] Terakawa A. , Natsume A. , Okada A. , Nishihata S. , Kuse J. , Tanaka K. , Takenaka S. , Ishikawa S. , and Yoshida K. I. , Bacillus Subtilis 5-Nucleotidases With Various Functions and Substrate Specificities, BMC Microbiology. (2016) 16, no. 1, 10.1186/s12866-016-0866-5, 27784292. PMC5080769 27784292

[59] Capra J. A. and Singh M. , Predicting Functionally Important Residues From Sequence Conservation, Bioinformatics. (2007) 23, no. 15, 1875–1882, 10.1093/bioinformatics/btm270. 17519246

[60] Russell R. B. , Copley R. R. , and Barton G. J. , Protein Fold Recognition by Mapping Predicted Secondary Structures, Journal of Molecular Biology. (1996) 259, no. 3, 349–365, 10.1006/jmbi.1996.0325. 8676374

[61] Sawal H. A. , Shad N. A. , Ejaz R. , and Aziz S. , Functional Categorization and Comparative 3D Model Study of TMEM16B, NUST Journal of Natural Sciences. (2023) 8, no. 2, 45–52.

[62] Szklarczyk D. , Kirsch R. , Koutrouli M. , Nastou K. , Mehryary F. , Hachilif R. , Gable A. L. , Fang T. , Doncheva N. T. , Pyysalo S. , Bork P. , Jensen L. J. , and von Mering C. , The STRING Database in 2023: Protein–Protein Association Networks and Functional Enrichment Analyses for Any Sequenced Genome of Interest, Nucleic Acids Research. (2023) 51, no. D1, D638–D646, 10.1093/nar/gkac1000, 36370105. 36370105 PMC9825434

[63] Shalayel M. H. , Al-Mazaideh G. M. , Aladaileh S. H. , Al-Swailmi F. K. , and Al-Thiabat M. G. , Vitamin D Is a Potential Inhibitor of COVID-19: In Silico Molecular Docking to the Binding Site of SARS-CoV-2 Endoribonuclease Nsp15, Pakistan Journal of Pharmaceutical Sciences. (2020) 33, no. 5, 2179–2186, 33824127. 33824127

